# Assessment of sensitization to grape and wine allergens as possible causes of adverse reactions to wine: a pilot study

**DOI:** 10.1186/s13601-015-0065-8

**Published:** 2015-06-22

**Authors:** Nadine Jaeckels, Iris Bellinghausen, Petra Fronk, Bärbel Heydenreich, Joachim Saloga, Heinz Decker

**Affiliations:** Institute for Molecular Biophysics, Johannes Gutenberg-University Mainz, Jakob-Welder-Weg 26, D-55128 Mainz, Germany; Department of Dermatology, University Medical Center Mainz, Langenbeckstraße 1, D-55131 Mainz, Germany

**Keywords:** Wine intolerance, LTP, Wine, CAST, CCD

## Abstract

**Background:**

In a recently performed survey with 4000 randomly selected persons, 68 (7.2 %) of 948 respondents reported intolerance and/or allergy-like symptoms to wine. The aim of this study was to analyze whether a real sensitization to wine proteins could be confirmed by diagnostic and/or immunological settings.

**Findings:**

For this purpose, 19 subjects with self-reported intolerance to wine of the invited subjects and 10 controls without a history of intolerance participated in an allergological examination (skin prick test, ImmunoCAP for determination of specific IgE antibodies, CAST for testing basophil activation, ImmunoBlot for testing specificity of IgE-antibodies). For the allergological work-up red and white grapes, selected wines, and the purified lipid transfer protein (LTP), a known grape allergen, were used. 7 subjects showed evidence of IgE sensitization to wine or grape extracts, including one control. One participant with symptoms of intolerance showed a positive skin prick test to red grape, a positive ImmunoCAP to grape, a positive cellular antigen stimulation test (CAST) and inhibition of Western blot by removal of cross-reactive carbohydrate determinants (CCD).

**Conclusion:**

The presented study focused on the grape protein-related IgE-mediated cause of intolerance to wine (true allergy) and not on other wine components or fining agents (other forms of intolerance). A sensitization to grape and wine proteins was observed in our cohort. In one case, this reactivity could be explained by cross-reactivity to CCD. The results of this pilot study need to be validated in greater cohorts.

## Introduction

Since thousands of years wine is known as popular alcoholic beverage. It consists of many ingredients produced by the grape or microorganism during the process of wine production [1]. Proteins are minor components in wine; some of them are discussed to be potential allergens. Especially in the Mediterranean area some cases of intolerance have been reported after consumption of/ or contact with grapes and their products [2–10]. A part of the studies focused on the proteins as cause for the reactions. As a result of these studies lipid transfer proteins (LTP), endochitinase 4 and thaumatin-like proteins (TLP) were discussed as potential allergens in grapes and wine [2]. However, Vassilopoulou et al. [3] reported that other grape proteins may be involved as well in the sensitization such as β-1,3-glucanase while TLPs and chitinases did not function as allergens. During the wine making process grape proteins are exposed to various conditions like high alcohol content or very low pH. Thus, a question arises whether LTP has still an allergenic potential after the wine making process. Investigations on the structural stability of LTP from grapes and those of wine did not elucidate obvious changes. As a consequence LTP in wine may still act as an allergen [11]. Based on an earlier epidemiological study “prevalence of wine intolerance” in our area 7.2 % of 948 participants reported intolerance symptoms after consuming grapes or wine [12]. The present study focuses on proteins derived from grapes and their allergenic potential, especially on LTP, which is listed as acknowledged allergen (www.allergen.org). For this purpose some participants of the earlier epidemiologic study underwent detailed allergological testing.

## Methods

### Study subjects

Twenty three women and six men aged 22 to 63 participated in the study. The mean age was 40, median 39 years. Of these, 19 persons reported wine intolerance (Table 1, No. 1–19). The ten remaining persons served as control group (No. 20–29). All participants were further asked about their symptoms after wine consumption (among others). The study was approved by the local ethics committee (reference number: 837.194.11(7736); approved: 22.06.2011). Informed consent was obtained from all subjects before the study.

**Table 1:** Summary of allergological investigations (n = 29)

Participant	Age	Gender	Prick test										ImmunoCAP			CAST	ImmunoBlot	Symptoms after wine consumption	Further self-reported intolerances
			Solvent	10 mg/ml Histamine	Rhinehessen Dornfelder	Rhinehessen Riesling	Moselle Pinot gris	Moselle Pinot noir	Moselle Riesling	red grape	white grape	red grape juice	total IgE [kU/l]	f259 Grape [kU/l]	CAP class				
1	58	m	no prick test										53.2	0	0	/	-	D, R, SC, T	histamine intolerance
2	39	m	−/−	5/10	−/−	−/−	4/12	2/2	4/12	3/6	−/−	−/−	192	0.01	0	neg	-	R, AS	pollen, house-dust, apple, wine, cats
3	56	f	1/2	8/30	−/−	−/−	−/−	−/−	−/−	−/−	−/−	−/−	314	0.02	0	/	-	BS, SW	house-dust, drugs, nuts, grape, wine
4	60	f	−/−	5/7	5/6	1/2	2/3	2/2	5/7	0/1	0/1	2/3	5.38	0	0	neg	-	I, F, SR, D	drugs
5	35	m	−/−	5/8	−/−	−/−	−/−	−/−	−/−	−/−	−/−	−/−	286	0.05	0	neg	-	SW	house-dust
6	41	f	−/−	5/20	−/−	−/−	−/−	−/−	−/−	−/−	−/−	−/−	22	0	0	neg	-	AS, H	lactose, pollen, grass, rye
7	23	f	−/−	5/20	−/−	−/−	−/−	−/−	−/−	3/3	1/1	−/−	910	1.26	2	pos	+++	I, SR, H	pollen, house-dust, strawberries, apple, plum, peach,kiwi, carrot, wine
8	60	f	−/−	+++	+	+	+	−/−	+	−/−	−/−	+	10.4	0	0	neg	-	H	pollen, grapes, wine
9	42	f	−/−	5/15	−/−	−/−	−/−	−/−	−/−	−/−	−/−	−/−	10.6	0	0	/	-	V, D, SC, BP, CC, H	nickel
10	58	f	−/−	5/20	−/−	−/−	−/−	−/−	−/−	−/−	−/−	−/−	222	0.02	0	neg	-	R, H	alcohol, wine, beer
11	41	f	−/−	5/20	−/−	−/−	−/−	−/−	−/−	−/−	−/−	−/−	10.7	0.01	0	neg	-	n.d.	n.d.
12	54	f	−/−	10/30	−/−	−/−	−/−	−/−	−/−	−/−	−/−	−/−	11.9	0.03	0	/	-	R, BS, H	/
13	24	f	no prick test										189	0.01	0	pos	-	I, F	pollen
14	30	f	−/−	5/20	−/−	−/−	−/−	−/−	−/−	−/−	−/−	−/−	74.3	0	0	neg	-	D, T, H	/
15	30	f	−/−	10/15	−/−	−/−	−/−	−/−	−/−	−/−	−/−	+	109	0.05	0	pos	-	I, R	pollen, house-dust, nuts, kiwi, sweet pepper, seafood
16	50	f	−/−	10/30	2/3	2/3	2/3	3/5	3/4	2/3	1	8/15	359	0.01	0	neg	-	n.d.	wine
17	41	f	−/−	++	−/−	−/−	−/−	−/−	−/−	−/−	−/−	−/−	6.91	0.02	0	neg	-	F, D, SC, H	wine
18	28	f	−/−	6/22	−/−	−/−	−/−	−/−	−/−	−/−	−/−	−/−	214	0	0	neg	/	D	fructose intolerance, house-dust
19	24	m	−/−	5/25	−/−	−/−	−/−	−/−	−/−	−/−	−/−	−/−	95.4	0	0	pos	/	SR, H	pollen
20	45	f	no prick test										108	0	0	neg	/	/	pollen, house-dust, oysters
21	34	f	−/−	++	−/−	−/−	−/−	−/−	−/−	−/−	−/−	−/−	16.1	0	0	neg	-	/	/
22	63	m	−/−	++	−/−	−/−	−/−	−/−	−/−	−/−	−/−	−/−	622	0.05	0	neg	-	/	canned mushrooms
23	30	f	−/−	5/25	−/−	−/−	−/−	−/−	−/−	−/−	−/−	−/−	2.58	0	0	neg	+	/	/
24	60	f	−/−	7/20	−/−	−/−	−/−	−/−	−/−	2/4	3/5	−/−	40.7	0	0	pos	/	/	pollen, nuts, apple
25	29	f	−/−	6/25	−/−	−/−	−/−	−/−	−/−	−/−	−/−	−/−	35.7	0	0	neg	/	/	pollen
26	27	f	−/−	5/20	−/−	−/−	−/−	−/−	−/−	−/−	−/−	−/−	12.1	0	0	neg	/	H	/
27	28	m	−/−	3/15	−/−	−/−	−/−	−/−	−/−	−/−	−/−	−/−	109	0	0	neg	/	/	/
28	22	f	−/−	8/20	−/−	−/−	−/−	−/−	−/−	−/−	−/−	−/−	114	0	0	neg	/	/	/
29	28	f	−/−	7/22	−/−	−/−	−/−	−/−	−/−	−/−	−/−	−/−	90.5	0	0	neg	/	/	/

Subject group with self-reported wine intolerance: *No.1–19* control group: No. 20–29; *m* male; *f* female; *neg* negativ; *pos* positiv; +++ large reaction (size of histamine reaction); ++: normal reaction, +: weak reaction; −: no reaction (wheal diameter < 2 mm); /: no test performed; CAP classes for specific IgE antibodies: 0: < 0.35kU/l; 1: 0.35–0.7 kU/l: 2: 0.7–3.5 kU/l; 3: 3.5–17.5 kU/l; 4: 17.5–50 kU/l; 5: 50–100 kU/l; 6: >100 kU/l; *n.d*. no data; *H* headaches; *D* diarrhea; *R* rhinorrhea; *I* itching; *F* flushed skin; *SR* skin rash, hives, edema; *SC* stomach or intestinal cramps; *BS* burning sensation in lips, palate, neck; *SW* swelling of lips, mouth, throat; *AS* shortness of breath/asthma; *T* tachycardia; *V* vomiting; *BP* low blood pressure; *CC* circulatory collapse. Prick test results are given in wheal diameter/ flush diameter, in mm (x/y). Moselle Riesling: Bernkasteller Badstube, Staatsweingut Mosel, Bernkastel-Kues, Germany, 2009, 11 %vol; Moselle Pinot gris: Grauburgunder, Staatliche Weinbaudomäne Trier, Germany, 12,5 %vol, 2009; Moselle Pinot noir: Avelsbacher Hammerstein Spätburgunder, Staatliche Weinbaudomäne Trier, Germany, 12 %vol, 2009; Rhinehessen Riesling: Riesling, Winery of the City of Mainz, Mainz, Germany, 12,5 %vol, 2009; Rhinehessen Dornfelder: Dornfelder, Winery of the City of Mainz, Mainz, Germany, 13,5 %vol, 2009

### Allergological investigations

Clinical interventions were conducted at the Department of Dermatology of the University Medical Center Mainz, Germany. History was taken and prick test was performed with various grapes and grape products (Table 1).

Total serum IgE and allergen-specific IgE (ImmunoCAP product code for grape: f259, CCD/MUXF3: o214, grass: gx1, birch: t3) were determined applying the ImmunoCAP® test (Phadia AB, Thermo Fisher Scientific, Waltham, Massachusetts, USA). In some cases, serum was preincubated for 30 min with 20 μg/ml ProGlycAn CCD-blocker/inhibitor (purified MUXF structure of digested bromelain, kindly provided by Prof. W. Aberer, ProGlycAn, Vienna, Austria) [13].

Release of sulfidoleukotriens after allergen stimulation (purified LTP of Dornfelder red wine: 640 pg/ml - 10 μg/ml) was investigated by CAST (Cellular Allergen Stimulation Test, Bühlmann Laboratories AG, Schönenbuch, Switzerland) according to the instructions of the manufacturer.

### Biochemical investigations

The reactivity of serum IgE Ab to various proteins was examined by ImmunoBlots. Therefore, SDS-PAGE (homemade 12.5 % and 15 % polyacrylamide gels pH 8.8 with 3 % stacking gels pH 6.8) of the various samples (Fig. 2) was performed as described before [11]. After electrophoresis the proteins were transferred on a nitrocellulose membrane by semi-dry blotting. Nonspecific binding sites were blocked using 3 % BSA. Following this, the membrane was incubated with serum, bound IgE Ab were detected using HRP-conjugated goat anti-human IgE pAb (Invitrogen, Life Technologies GmbH, Darmstadt, Germany). In all experiments, a corresponding Coomassie-stained SDS-PAGE with the same samples served as control. To study the influence of the cross-reactive carbohydrates determinants (CCD) part of these experiments were performed with previous incubation of serum with 20 μg/ml CCD-blocker [13].

## Findings

### Symptoms

**Fig. 1:**
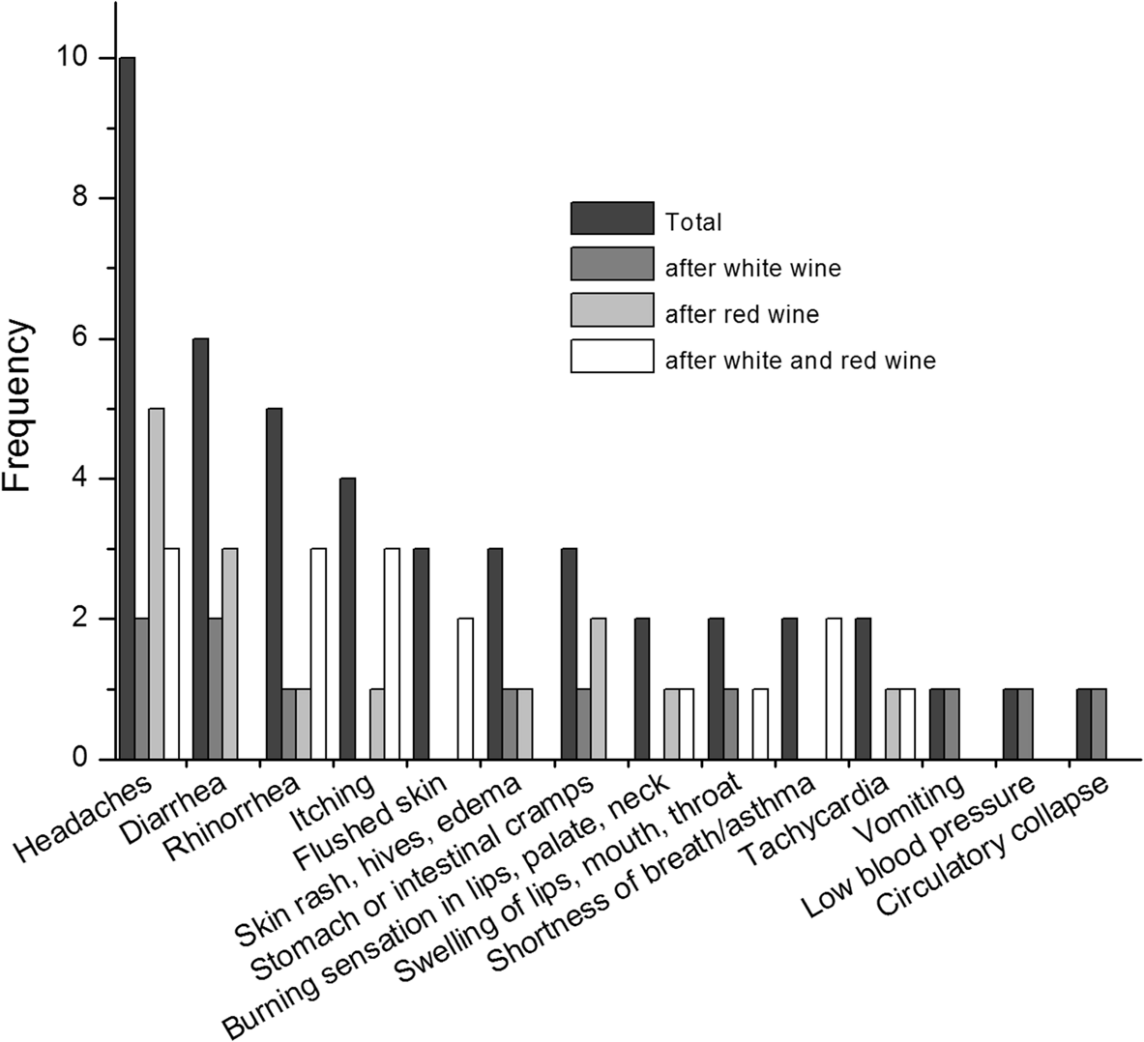
Frequency of intolerance symptoms after wine consumption (n = 29). Symptoms after white-, red-, or white and red wine consumption were examined

The most frequently reported symptoms after wine consumption of the 19 participants with self-reported wine intolerance were in accordance with our former study [12] (rhinorrhea, itching etc., Fig. 1). The participants stated that adverse reactions could be observed immediately up to 2 h after consumption of grape products.

### Allergy testing

Prick test was conducted with 26 participants. Seven persons showed a positive reaction (wheal of at least 3 mm diameter) towards any grape product (Table 1). Two of these persons reacted only towards red or white grapes (No. 7, 24) and one person to red grape juice (No. 15). The other three persons reacted to grapes and wine (No. 2, 8, 16) and only one person only to wine (No. 4) (Table 1).

Total IgE was above range in 13 participants (9 intolerant, 4 controls). Four of these also showed a positive reaction in prick test (No. 2, 7, 15, 16). Only one person showed specific sensitization with ImmunoCAP (grape: CAP class 2). This participant (No. 7) also suffered from severe allergy to pollen (grass: CAP class 6; birch: CAP class 5), and reacted towards CCD (MUXF3: CAP class 3). A preincubation of serum taken from the same person at a later time point with CCD-blocker led to a reduction of the concentration of MUXF3-specific IgE (2.06 kU/l before, 0.25 kU/l after) as well as of grape-specific IgE (0.5 kU/l before, 0.04 kU/l after) while birch-specific IgE was not reduced after CCD blockade (91.2 kU/l before, 96.1 kU/l after).

In addition, CAST was performed with isolated leucocytes from 25 persons to analyze basophil activation. Release of sulfidoleukotrienes by basophil granulocytes was measured after stimulation with varying concentrations of purified LTP. Overall, basophil granulocytes of five participants (No. 7, 13, 15, 19, 24) reacted with increased leukotriene release (sulfidoleukotriene concentration > 200 pg/ml). Thus, a relationship of CAST and prick test could be found since three (2 intolerant No. 7, 15; one control No. 24) of these five persons also showed a positive prick test.

**Fig. 2:**
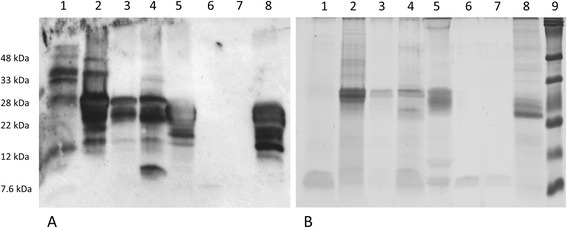
ImmunoBlot of IgE antibody reactivity applying different grape and wine proteins. **a** ImmunoBlot of participant No. 7, who showed positive reactions in all tests; **b** Coomassie-stained 15 % SDS-PAGE. Samples: 1: peach; 2: red grapes; 3: Dornfelder grapes (winery Fleischer, Mainz); 4: white grapes; 5: Dornfelder wine (after PVP precipitation); 6: purified LTP from Dornfelder wine [15]; 7: purified LTP from Dornfelder grapes [15]; 8: Riesling wine (after Lyophilisation); 9: RunBlue Dual Color SDS marker (Expedeon, Cambridge,United Kingdom). For SDS-PAGE 30 μl of the extract, which was treated 3:1 with SDS sample buffer and incubated 95 °C for 10 min, was applied

**Fig. 3:**
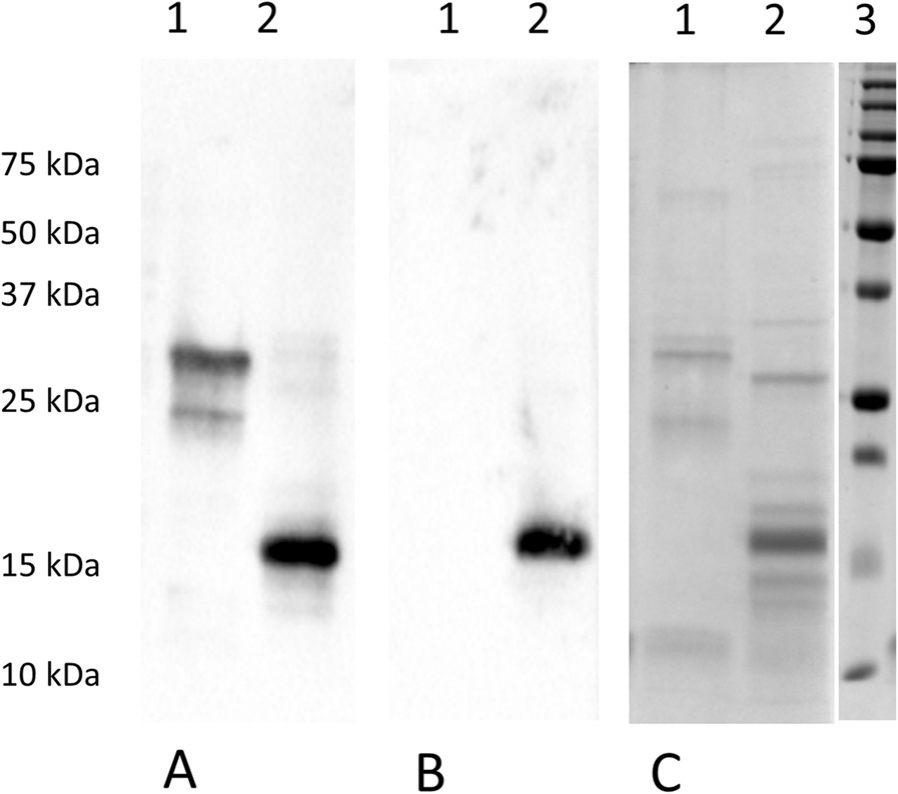
ImmunoBlot of IgE antibody reactivity applying grape and birch extracts after inhibition of the CCD specific IgE antibodies. **a** ImmunoBlot control of patient No. 7. **b** ImmunoBlot after preincubation of serum with 20 μg/ml CCD-blocker. **c** Coomassie-stained 12.5 % SDS-PAGE. Samples: 1: white grapes; 2: birch; 3: Precision Plus Protein dual Color Standard (BioRad, Munich, Germany)

For participant No. 7 all tests were positive (total IgE 910 kU/l, grape: CAP class 2, birch: CAP class 5, MUXF3. CAP class 3). This person also showed a strong reaction towards different grape and wine proteins in the ImmunoBlot. Besides a severe reaction of IgE Ab to grape and wine proteins a reaction to peach could be detected, while a reaction to LTP was hardly detectable (Fig. 2). After incubation of the serum taken from the same person at a later time point with CCD-blocker (purified MUXF structure of digested bromelain) the reactivity against grape proteins completely disappeared, while a strong reactivity to a birch protein of nearly 17 kDa (Bet v 1) could still be detected (Fig. 3), this is in accordance to the ImmunoCAP results. The serum of participant No. 23 showed a weak ImmunoBlot, but the history of this person showed no evidence for intolerances. Sera from the other tested participants showed no reaction in ImmunoBlot experiments.

## Discussion

Some of the participants showed a sensitization towards grapes and wine in particular tests. A positive reaction in all tests was only observed in one participant (No. 7). For this participant a strong reaction of IgE Ab towards different proteins of grape and wine samples could be detected, especially to proteins with a molecular weight of 20–30 kDa (TLP, chitinases), which are discussed as allergens [2, 3, 7].

Due to the strong sensitization of this participant also to birch and grass pollen, pollen-associated cross reactions to food or CCD (MUXF3) might be the reason for these observed reactions [14, 15]. Inhibition experiments with CCD-blocker confirmed the latter assumption. However, a reaction to LTP (9–12 kDa) which was only detectable in the CAST indicated that some of the non-glycosylated wine proteins may also have an allergenic potential.

Our study focused on grape and wine proteins, especially LTP as known grape allergen. Nonetheless, intolerance to wine might be triggered by many other wine ingredients such as biogenic amines [16–18], acids and sulfite (sulfite declaration is mandatory for wine) [17], alcohol [7, 19, 20], or fining agents [21, 22]. Symptoms as flush and tachycardia might be explained by biogenic amines or even alcohol affecting the cardiovascular system by acting as vasodilator [19, 20]. The multitude of wine ingredients, especially in combination, could be responsible for intolerance reactions [1, 23].

## Conclusion

Nineteen persons with self-reported wine intolerance were tested for specific allergic reactions to wine. A sensitization to LTP correlating with a positive prick test could be observed in two participants of the subject group with self-reported intolerance and in one person of the control group. The strong sensitization to other grape and wine proteins of one of these participants could be explained by CCD-cross reactivity.

This pilot study demonstrates that there may be sensitizations to grape or wine proteins in the German wine drinking region of Mainz. In a further study this sensitization has to be proven in a larger number of participants to achieve a statistical validation. Additionally, other forms of intolerance should be considered, including histamine and sulphide.

## Abbreviations

Ab: Antibodies
BSA: Bovine serum albumin
CAST: Cellular allergen stimulation test
CCD: Cross reactive carbohydrate determinants
HRP: Horseradish peroxidase
IgE: Immunoglobulin E
LTP: Lipid transfer protein
pAb: polyclonal Antibody
PAGE: Polyacrylamide gel electrophoresis
SDS: Sodium dodecyl sulfate
TLP: Thaumatin-like protein
